# Supplementary material for: Predicting onset of symptomatic Alzheimerʼs disease with plasma p-tau217 clocks
Source: Nat Med. 2026 Feb 19;32(3):1085–94. doi: 10.1038/s41591-026-04206-y (PMC13004683; doi:10.1038/s41591-026-04206-y)
Supplement: Supplementary file 1 — Nine supplementary tables, 13 supplementary figures and captions for six supplementary videos. [file 41591_2026_4206_MOESM1_ESM.pdf]

nature medicine

Supplementary information https://doi.org/10.1038/s41591-026-04206-y

Predicting onset of symptomatic Alzheimer' s disease with plasma p-tau217 clocks

In the format provided by the authors and unedited

Supplementary information

Predicting onset of symptomatic Alzheimer disease with a plasma %p-tau217 clock

by Petersen *et al.*

**Reference**	Reference for positivity thresholds
**Consortium members**	Foundation for the National Institutes of Health (FNIH) Biomarkers Consortium Plasma Aβ and Phosphorylated Tau as Predictors of Amyloid and Tau Positivity in Alzheimer's Disease Project Team

Supplemental tables

Supplementary Table 1. Characteristics of longitudinal plasma %p-tau217 cohort.
Supplementary Table 2. Characteristics of individuals used to develop models of symptom onset.
Supplementary Table 3. Models of the age at AD symptom onset based on the estimated age at plasma %p-tau217 positivity.
Supplementary Table 4. Model diagnostics for linear regression models predicting AD symptom onset from estimated age of plasma %p-tau217 positivity.
Supplementary Table 5. Models of the age at AD symptom onset based on the estimated age at plasma %p-tau217 positivity including covariates.
Supplementary Table 6. Characteristics table for participants who were cognitively unimpaired at baseline, with at least one plasma %p-tau217 sample, and longitudinal clinical assessments.
Supplementary Table 7. Characteristics table for participants with at least one plasma %p-tau217 sample and longitudinal clinical assessments.
Supplementary Table 8. Clinical diagnoses in the Knight ADRC cohort.
Supplementary Table 9. Clinical diagnoses in the ADNI cohort.

Supplemental figures

Supplementary Figure 1. Comparison of plasma %p-tau217 clock models using restricted and unrestricted data ranges.
Supplementary Figure 2. Estimated age of %p-tau217 positivity using different clock models.
Supplementary Figure 3. Estimated age of %p-tau217 positivity in different cohorts.
Supplementary Figure 4. Probability of remaining cognitively unimpaired from AD is related to age at plasma %p-tau217 positivity.
Supplementary Figure 5. Probability of remaining cognitively unimpaired from AD is related to age at plasma %p-tau217 positivity.
Supplementary Figure 6. Models for time from plasma %p-tau217 positivity to symptom onset based on estimated age at plasma %p-tau217 positivity.
Supplementary Figure 7. Models for age at symptom onset based on estimated age at plasma %p-tau217 positivity.
Supplementary Figure 8. Clinical diagnosis as a function of estimated age at plasma %p-tau217 positivity and years from %p-tau217 positivity for initially cognitively unimpaired individuals.
Supplementary Figure 9. Relationships between cognitive impairment and %p-tau217 positivity by SILA using the Knight ADRC dataset.
Supplementary Figure 10. Relationships between cognitive impairment and %p-tau217 positivity by TIRA using the ADNI dataset.

Supplementary Figure 11. Relationships between cognitive impairment and %p-tau217 positivity by SILA using the ADNI dataset.
Supplementary Figure 12. C2N Diagnostics' PrecivityAD2 plasma p-tau217 and symptom onset models based on estimated years from p-tau217 positivity.
Supplementary Figure 13. Fujirebio Diagnostics' Lumipulse plasma p-tau217 and symptom onset models based on estimated years from p-tau217 positivity.

Supplementary videos

Supplementary Video 1. Animation of C2N Diagnostics' PrecivityAD2 %plasma p-tau217 trajectories transitioning between plots versus age and years since plasma positivity.
Supplementary Video 2. Animation of C2N Diagnostics' PrecivityAD2 plasma p-tau217 trajectories transitioning between plots versus age and years since plasma positivity.
Supplementary Video 3. Animation of Fujirebio Diagnostics' Lumipulse plasma p-tau217/Aβ42 trajectories transitioning between plots versus age and years since plasma positivity.
Supplementary Video 4. Animation of Fujirebio Diagnostics' Lumipulse plasma p-tau217 trajectories transitioning between plots versus age and years since plasma positivity.
Supplementary Video 5. Animation of Janssen's LucentAD Quanterix plasma p-tau217 trajectories transitioning between plots versus age and years since plasma positivity.
Supplementary Video 6. Animation of ALZpath's Quanterix plasma p-tau217 trajectories transitioning between plots versus age and years since plasma positivity.

Reference for positivity thresholds:

1 Schindler, S. E. *et al.* Head-to-head comparison of leading blood tests for Alzheimer's disease pathology. *Alzheimer's & dementia : the journal of the Alzheimer's Association* **20**, 8074-8096, doi:10.1002/alz.14315 (2024).

Foundation for the National Institutes of Health (FNIH) Biomarkers Consortium Plasma Aβ and Phosphorylated Tau as Predictors of Amyloid and Tau Positivity in Alzheimer's Disease Project Team

Anthony W. Bannon[1], Michael Baratta[2], Janaky Coomaraswamy[2], Jeffrey L. Dage[3.4], Iwona Dobler[2], Lei Du-Cuny[5], Kyle Ferber[6], John Hsiao (NIA)[7], Hartmuth Kolb[8], Emily A. Meyers[9], Yulia Mordashova[1], William Z. Potter[10], Maria Quinton[1], Dave Raunig[2], Erin G. Rosenbaugh[11], Carrie E. Rubel[6], Ziad S. Saad[8], J. Martin Sabandal[11], Patricia Saletti[12], Suzanne E. Schindler[13-15], Leslie M. Shaw[16], Gallen Triana-Baltzer[8], Christopher Weber[9], Henrik Zetterberg[17-21]

1. AbbVie, 1400 Sheridan Rd., North Chicago, IL 60064, USA
2. Takeda Pharmaceutical Company Ltd., 650 E Kendall St., Cambridge, MA 02142, USA
3. Department of Neurology, Indiana University School of Medicine, 355 W 16th St., Indianapolis, IN 46202, USA
4. Stark Neurosciences Research Institute, Indiana University School of Medicine, NB Building, 320 W 15th St #414, Indianapolis, IN 46202, USA
5. AbbVie Deutschland GmbH & Co. KG, Knollstraße 50, 67061, Ludwigshafen am Rhein, Rheinland-Pfalz, Germany
6. Biogen, 225 Binney St., Cambridge, MA 02142, USA
7. National Institute on Aging, 9000 Rockville Pike, Bethesda, MD 20894, USA
8. Precision Measures, Johnson & Johnson, 3210 Merryfield Row, San Diego, CA 20892, USA
9. Alzheimer's Association, 225 N Michigan Ave., Chicago, IL 60603, USA
10. Highly qualified expert
11. The Foundation for the National Institutes of Health, 11400 Rockville Pike, Suite 600, North Bethesda, MD 20852, USA
12. Alzheimer's Drug Discovery Foundation, 57 W 57th St #904, New York, NY 10019, USA
13. Department of Neurology, Washington University in St. Louis, 660 S. Euclid Ave., St. Louis, MO 63110, USA
14. Knight Alzheimer Disease Research Center, 4488 Forest Park Avenue, Suite 200, St. Louis, MO 63108, USA
15. Hope Center for Neurologic Diseases, 4370 Duncan Ave., St. Louis, MO 63110, USA
16. Department of Pathology and Laboratory Medicine, Perelman School of Medicine, University of Pennsylvania, 3400 Spruce St., Philadelphia, PA 19104, USA
17. Institute of Neuroscience and Physiology, Department of Psychiatry and Neurochemistry, The Sahlgrenska Academy at University of Gothenburg, Biskopsbogatan 27, S-431 80, Mölndal, Sweden
18. Clinical Neurochemistry Laboratory, Sahlgrenska University Hospital, Biskopsbogatan 27, S-431 80, Mölndal, Sweden
19. UK Dementia Research Institute Fluid Biomarkers Laboratory, UK DRI at UCL, 3rd Floor, Queen Square House, Queen Square, London WC1N 3BG, United Kingdom
19. Department of Neurodegenerative Disease, UCL Queen Square Institute of Neurology, Queen Square, London WC1N 3BG, United Kingdom
20. Hong Kong Center for Neurodegenerative Diseases, Clear Water Bay, Units 1501-1502, 1512-1518, 15/F Building 17W, 17 Science Park W Ave, Science Park, Hong Kong, China
21. Wisconsin Alzheimer's Disease Research Center, University of Wisconsin School of Medicine and Public Health, University of Wisconsin-Madison, 600 Highland Ave J5/1Mezzanine, Madison, WI 53792, USA

Supplementary Table 1. Characteristics of longitudinal plasma %p-tau217 cohort. Individuals were included who had two or more plasma %p-tau217 measurements at least one year apart. Values are presented as the median (interquartile range) for continuous variables and n (%) for categorical variables. The significance of differences between cohorts was evaluated by Chi-square tests for categorical values and Wilcoxon ranked sum tests for continuous values.

	**Full Cohort**		**Knight ADRC**		**ADNI**		
**Characteristic**	**N=**	**Values**	**N=**	**Values**	**N=**	**Values**	**p=**
**Age (Years)**	912	69.8 (65.0-75.0)	506	67.7 (61.7-72.4)	406	72.7 (67.9-78.0)	<0.0001
**Sex (% Female)**	912	474, 52%	506	274, 54.2%	406	200, 49.3%	0.10
***APOE* ε4 carriers (% carrier)**	912	320, 35.1%	506	181, 35.8%	406	139, 34.2%	0.60
**Years of Education**	912	16 (15-18)	506	16 (15-18)	406	16 (14-18)	0.88
**Cognitive impairment (% CDR>0)**	912	239, 26.2%	506	43, 8.5%	406	196, 48.3%	<0.0001
**CDR Sum of Boxes**	912	0 (0-0.5)	506	0 (0-0)	406	0.5 (0-1)	<0.0001
**Time from first to last plasma sample (Years)**	912	6.0 (4.1-8.6)	506	7.1 (5.0-11.0)	406	5.0 (4.0-6.5)	<0.0001
**Number of %p-tau217 values included**	912	3 (3-3)	506	3 (2-4)	406	3 (3-3)	0.02975
**First %p-tau217 included**	895	1.67 (0.62-3.94)	506	0.96 (0.19-2.41)	389	2.77 (1.53-5.71)	<0.0001

Supplementary Table 2. Characteristics of individuals used to develop models of symptom onset. Individuals were included who were initially cognitively unimpaired but diagnosed with cognitive impairment due to AD at their last assessment and who developed symptoms after estimated plasma %p-tau217 positivity.

	**Knight ADRC**		**ADNI**	
	**TIRA**	**SILA**	**TIRA**	**SILA**
**Number of individuals**	59	61	20	22
**Sex (% female)**	30 (50.8%)	30 (49.2%)	10 (50.0%)	11 (50.0%)
***APOE* ε4 carriers (% carrier)**	29 (49.2%)	29 (47.5%)	8 (40.0%)	8 (36.4%)
**Years of education**	16 (14-18)	16 (14-18)	16 (14.5-19.25)	16 (15-18.75)
**Estimated age of %p-tau217 positivity (years)**	71.0 (66.4-76.5)	72.2 (67.8-78.1)	67.5 (63.4-73.5)	73.7 (70.0-77.5)
**Clinical follow-up time (years)**	13.2 (9.0-14.6)	13.0 (9.0-14.3)	10.1 (8.4-11.4)	10.1 (8.6-11.9)
**Clinical assessments (n)**	11 (7.5-13)	11 (8-13)	10.5 (9-12.25)	10.5 (9-12.75)
**Age (years)**	73.1 (67.0-79.3)	73.1 (67.5-79.2)	73.3 (71.7-77.2)	73.2 (71.2-77.0)
**Baseline visit to estimated age of positivity (years)**	1.2 (-2.8-5.3)	0.3 (-4.1-3.8)	7.7 (1.9-11.3)	2.1 (-4.2-3.7)

Supplementary Table 3. Models of the age at AD symptom onset based on the estimated age at plasma %p-tau217 positivity. Linear regression coefficient and model fit metrics are reported. In all models, Estimated age at %p-tau217 positivity was significant. Linear regression coefficient and model fit metrics are reported.

		TIRA								SILA							
		Knight ADRC				ADNI				Knight ADRC				ADNI			
		Beta	SE	t-value	p	Beta	SE	t-value	p	Beta	SE	t-value	p	Beta	SE	t-value	p
Coef.	(Intercept)	37.41	4.724	7.92	<0.001	54.21	8.243	6.58	<0.001	35.29	4.743	7.44	<0.001	41.25	9.026	4.57	<0.001
	Estimated age at %p-tau217 positivity	0.61	0.066	9.35	<0.001	0.39	0.119	3.26	0.004	0.64	0.065	9.78	<0.001	0.54	0.122	4.43	<0.001
Model fit	Residual SE	4.362				4.583				4.164				3.926			
	Adj. R-squared	0.599				0.3366				0.612				0.470			
	F-statistic (p)	F(1,57)=87.49 (<0.001)				F(1,18)=10.64 (0.004)				F(1,59)=95.61 (<0.001)				F(1,20)=19.61 (<0.001)			

Supplementary Table 4. Model diagnostics for linear regression models predicting AD symptom onset from estimated age of plasma %p-tau217 positivity. Diagnostic tests were performed across all cohort-method combinations (Knight ADRC and ADNI using TIRA and SILA methods) to validate the use of linear modeling. Normality of residuals was confirmed using Shapiro-Wilk tests, homoscedasticity was verified through Breusch-Pagan tests, and linearity was supported by AIC-based model comparison for comparing regression models. F-tests comparing polynomial models to linear models showed no significant improvement from higher-order terms across all combinations.

Cohort	Method	Shapiro-Wilk p-value	Breusch-Pagan p-value	AIC Linear	AIC Quadratic	AIC Cubic	F-test p-value (Quadratic)	F-test p-value (Cubic)
Knight ADRC	TIRA	0.899	0.332	345.2	345.3	346.9	0.182	0.556
Knight ADRC	SILA	0.606	0.328	351.1	351.2	352.6	0.175	0.465
ADNI	TIRA	0.693	0.752	121.6	122.4	123.9	0.337	0.520
ADNI	SILA	0.332	0.200	126.5	126.6	128.4	0.200	0.690

Supplementary Table 5. Models of the age at AD symptom onset based on the estimated age at plasma %p-tau217 positivity including covariates. Linear regression coefficient and model fit metrics are reported. In all models, estimated age at %p-tau217 positivity was significant. Also, in all models, covariates were found to be not significant except *APOE* ε4 carrier status in the TIRA-Knight ADRC model. Additional analysis was performed on this model that found only a small proportion of unique variance determined by *APOE* ε4 carrier status (semi-partial $R^2 = 0.018$, partial eta squared = 0.05). Sex and years of education contributed even less unique variance (semi-partial $R^2 = 0.016$ and 0, respectively), meaning their inclusion did not meaningfully improve the model. Overall, these results suggest that while *APOE* ε4 carrier status may have a minor role in predicting age of symptom onset, the covariates offer little additional predictive value beyond estimated age of %p-tau217.

		**TIRA**								**SILA**							
		**Knight ADRC**				**ADNI**				**Knight ADRC**				**ADNI**			
		**Beta**	**SE**	**t-value**	**p**	**Beta**	**SE**	**t-value**	**p**	**Beta**	**SE**	**t-value**	**p**	**Beta**	**SE**	**t-value**	**p**
**Coefficients**	**(Intercept)**	42.43	6.564	6.46	<0.001	21.67	20.253	1.07	0.302	37.35	6.556	5.70	<0.001	19.65	16.445	1.20	0.248
	**Estimated age at %p-tau217 positivity**	0.57	0.072	7.88	<0.001	0.57	0.151	3.76	0.002	0.61	0.072	8.44	<0.001	0.68	0.145	4.70	<0.001
	**Female**	1.30	1.171	1.11	0.273	6.75	3.860	1.75	0.101	1.81	1.126	1.61	0.114	3.82	2.610	1.47	0.161
	***APOE* ε4 carrier**	-2.78	1.212	-2.30	0.026	3.47	2.414	1.44	0.171	-1.99	1.160	-1.71	0.092	2.99	1.977	1.51	0.148
	**Years of education**	-0.05	0.209	-0.26	0.800	0.96	0.652	1.47	0.163	-0.01	0.201	-0.05	0.958	0.50	0.456	1.09	0.291
**Model fit**	**Residual SE**	4.185				4.484				4.021				3.884			
	**Adj. R-squared**	0.630				0.3652				0.638				0.481			
	**F-statistic (p)**	F(4,54) = 25.74 (<0.001)				F(4,15)=3.732 (0.027)				F(4,56) = 27.45 (<0.001)				F(4,17) = 5.865 (0.0037)			

Supplementary Table 6. Characteristics table for participants who were cognitively unimpaired at baseline, with at least one plasma %p-tau217 sample, and longitudinal clinical assessments. General and baseline characteristics for longitudinal clinical assessments.

	**Knight ADRC**		**ADNI**	
	**TIRA**	**SILA**	**TIRA**	**SILA**
**Number of individuals**	686	682	199	198
**Sex (% Female)**	389 (56.7%)	386 (56.6%)	108 (54.3%)	107 (54.0%)
***APOE* ε4 carriers (% carrier)**	254 (37.4%)	253 (37.5%)	58 (29.1%)	58 (29.3%)
**Years of education**	16 (15-18)	16 (15-18)	16 (15-18)	16 (15-18)
**Estimated age of %p-tau217 positivity (years)**	79.14 (72.2-85.9)	77.0 (70.9-81.8)	82.1 (72.8-89.6)	79.1 (73.0-84.4)
**Clinical follow-up time (years)**	8.9 (5.2-14.1)	8.9 (5.2-14.1)	9.5 (7.2-11.3)	9.5 (7.1-11.3)
**Clinical assessments (n)**	8 (5-12)	8 (5-12)	9 (7-10.5)	9 (7-10.75)
**Age (years)**	68.1 (63.6-72.7)	68.1 (63.5-72.8)	71.9 (68.4-76.5)	71.7 (68.4-76.5)
**Baseline visit to estimated age of positivity (years)**	-12.1 (-18.9 to -4.2)	-9.7 (-14.4 to -3.8)	-10.7 (-18.1 to -0.1)	-8.1 (-11.8 to -1.7)
**CDR (0/0.5/1/2)**	686/0/0/0	682/0/0/0	199/0/0/0	198/0/0/0
**CDR-SB score**	0 (0-0)	0 (0-0)	0 (0-0)	0 (0-0)
**Number of progressors (n)**	82 (12.0%)	82 (12.0%)	28 (14.1%)	28 (14.1%)

Supplementary Table 7. Clinical diagnoses in the Knight ADRC cohort. Clinical diagnoses are shown across all assessments (n=6,736). The typical AD dementia syndrome category included individuals with a typical AD dementia syndrome (including amnestic, logopenic aphasia, posterior cortical dysfunction, or dysexecutive presentations) per clinician judgement as either as a sole diagnosis or as the primary diagnosis with other co-occurring disorders. The other dementia syndrome category encompassed individuals with uncertain etiology, questionable impairment, or specific diagnoses of other neurodegenerative conditions.

Total assessments (o=6736)	
Cognitively normal (o=5680, 84%)	
Typical AD dementia syndrome (o=632, 9%)	
	Typical AD dementia (o=561)
	Typical AD dementia with other disorders not contributing to dementia (o=71)
Other dementia syndrome: uncertain, atypical, or suspected non-AD syndrome (o=424, 6%)	
	Uncertain etiology (o=337)
	Uncertain etiology, possible non-AD dementia (o=25)
	Uncertain etiology, questionable impairment (o=25)
	Dementia with Lewy bodies or Parkinson disease dementia (o=17)
	Frontotemporal dementia (o=4)
	Vascular dementia (o=16)

Supplementary Table 8. Characteristics table for participants with at least one plasma %p-tau217 sample and longitudinal clinical assessments. General and baseline characteristics for longitudinal clinical assessments.

	Knight ADRC		ADNI	
	**TIRA**	**SILA**	**TIRA**	**SILA**
**Number of individuals**	830	825	371	370
**Sex (% Female)**	442 (53.3%)	439 (53.2%)	190 (51.2%)	190 (51.4%)
***APOE* ε4 carriers (% carrier)**	328 (39.9%)	326 (39.9%)	122 (32.9%)	122 (33.0%)
**Years of education**	16 (14-18)	16 (14-18)	16 (14-18)	16 (14-18)
**Estimated age of %p-tau217 positivity (years)**	78.5 (70.5-85.4)	76.5 (70.4-81.6)	80.7 (70.4-88.4)	77.7 (71.3-82.6)
**Clinical follow-up time (years)**	8.0 (5.0-14.0)	8.0 (5.0-14.0)	9.2 (6.2-11.3)	9.2 (6.2-11.3)
**Clinical assessments (n)**	7 (4-11)	7 (4-11)	9 (7-11)	9 (7-11)
**Age (years)**	68.7 (64.7-73.7)	68.7 (64.7-73.7)	71.0 (66.8-75.9)	71.0 (66.8-75.9)
**Baseline visit to estimated age of positivity (years)**	-11.1 (-18.2 to -1.5)	-8.9 (-13.7 to -1.8)	-10.6 (-18.1 to 3.0)	-7.6 (-11.4 to 0.1)
**CDR (0/0.5/1/2)**	686/128/15/1	682/127/15/1	199/171/1/0	198/171/1/0
**CDR-SB score**	0 (0-0)	0 (0-0)	0 (0-1)	0 (0-1)
**Number of progressors (n)**	82 (9.9%)	82 (9.9%)	28 (7.5%)	28 (7.6%)

Supplementary Table 9. Clinical diagnoses in the ADNI cohort. Clinical diagnoses are shown across all assessments (n=3,423). The typical AD dementia syndrome category included individuals with a typical AD dementia syndrome (including amnestic, logopenic aphasia, posterior cortical dysfunction, or dysexecutive presentations) per clinician judgement as either as a sole diagnosis or as the primary diagnosis with other co-occurring disorders. The other dementia syndrome category encompassed individuals with uncertain etiology, questionable impairment, or specific diagnoses of other neurodegenerative conditions.

Total assessments (o=3423)	
Cognitively normal (o=1861, 54%)	
Typical AD dementia syndrome (o=1041, 30%)	
	Typical AD dementia (o=850)
	Typical AD dementia primarily with other potential disorders (o=191)
Other dementia syndrome: uncertain, atypical, or suspected non-AD syndrome (o=521, 6%)	
	Uncertain etiology (o=489)
	Unspecified non-AD etiology (o=32)

Supplementary Figure 1. Comparison of plasma %p-tau217 clock models using restricted and unrestricted data ranges. Clock models relating plasma %p-tau217 time to plasma %p-tau217 levels are shown for the Knight ADRC (A) and ADNI (B) cohorts. Models were created using two approaches: TIRA (green) and SILA (orange). Solid lines represent clock models developed using the restricted data range (1.06% to 10.45% plasma %p-tau217) identified through variance analysis as having consistent rates of change, while dashed lines show models developed using the full unrestricted data range. The vertical black dashed line represents the plasma %p-tau217 positivity threshold of 4.06% (aligned with amyloid PET Centiloid value of 20), and the horizontal black dashed line represents time zero (estimated time of plasma %p-tau217 positivity). The decreasing green dashed line in (B) corresponds to the TIRA model for plasma values below the minimum value of the restricted range (1.06%) where variance is high, which supports the need to restrict models to the restricted range of values.

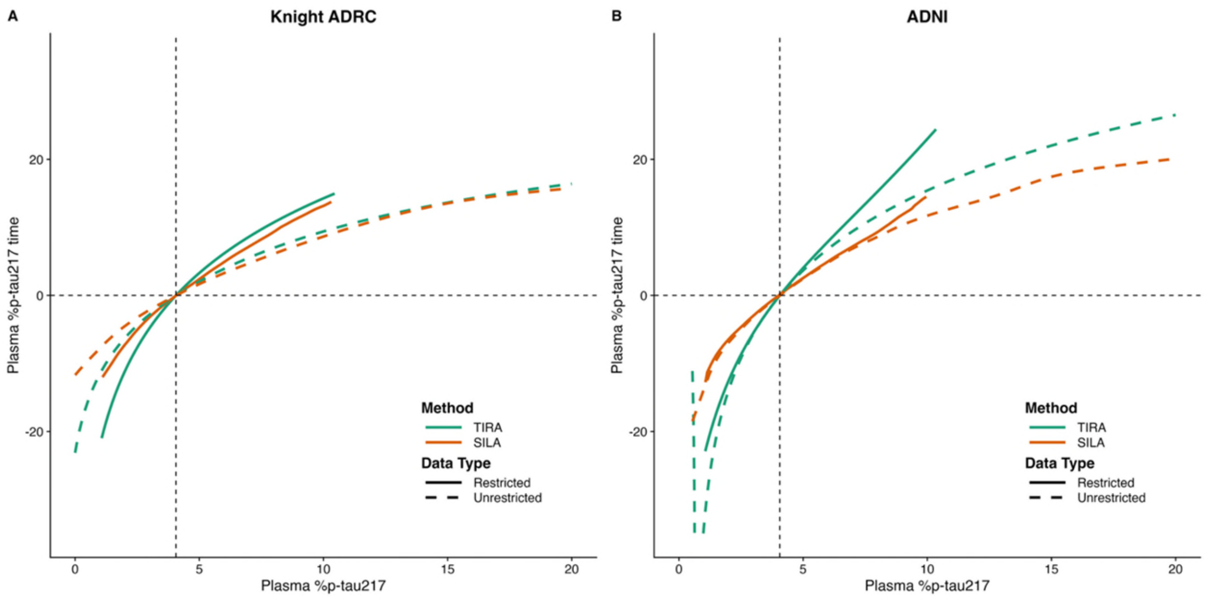

Supplementary Figure 2. Estimated age of %p-tau217 positivity using different clock models. Scatter plots show the associations in a cross-cohort comparison between estimated ages of %p-tau217 positivity based on models created using the TIRA (**A**) and SILA (**B**) approaches in the Knight ADRC and ADNI cohorts. Green points represent values from Knight ADRC participants; black points represent values from ADNI participants. The dashed red line indicates perfect agreement between models.

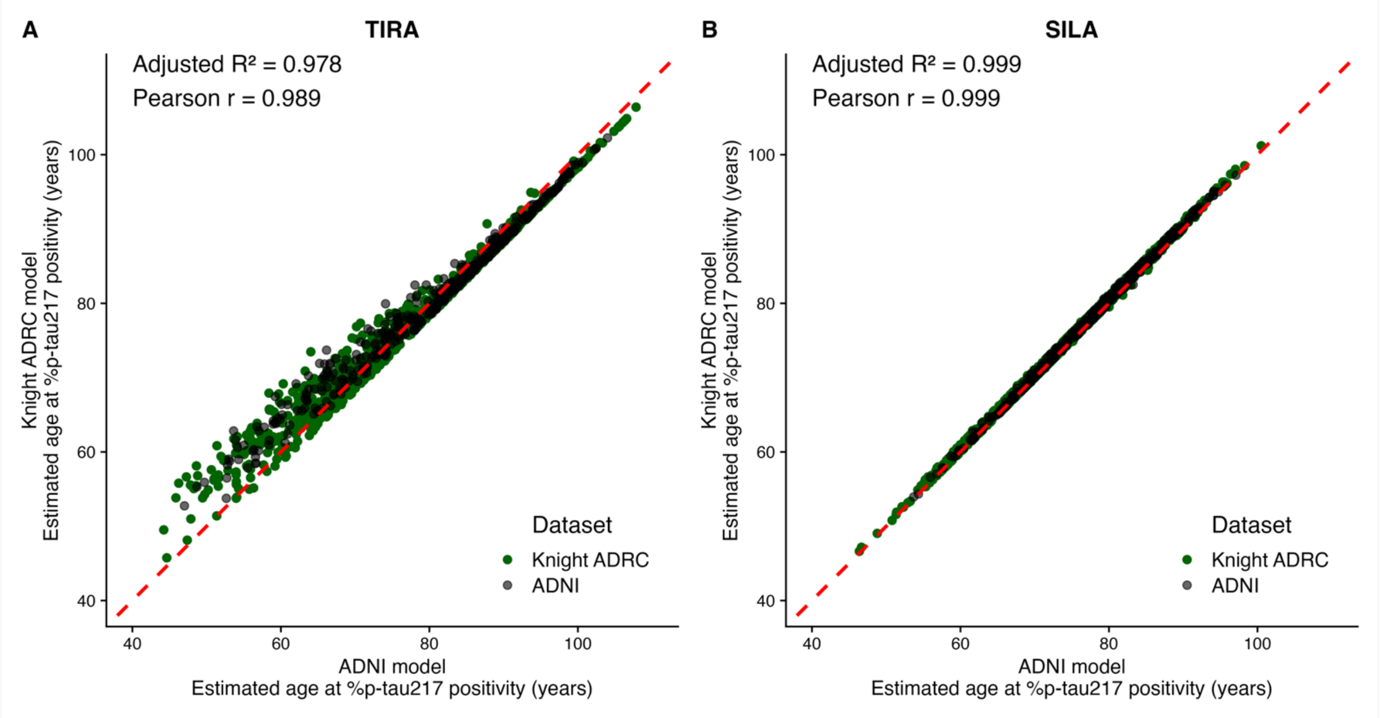

Supplementary Figure 3. Estimated age of %p-tau217 positivity in different cohorts. Scatter plots show the associations between estimated ages of %p-tau217 positivity based on models created using the TIRA and SILA approaches in the Knight ADRC (**A**) and ADNI (**B**) cohorts. The dashed red line indicates perfect agreement between models.

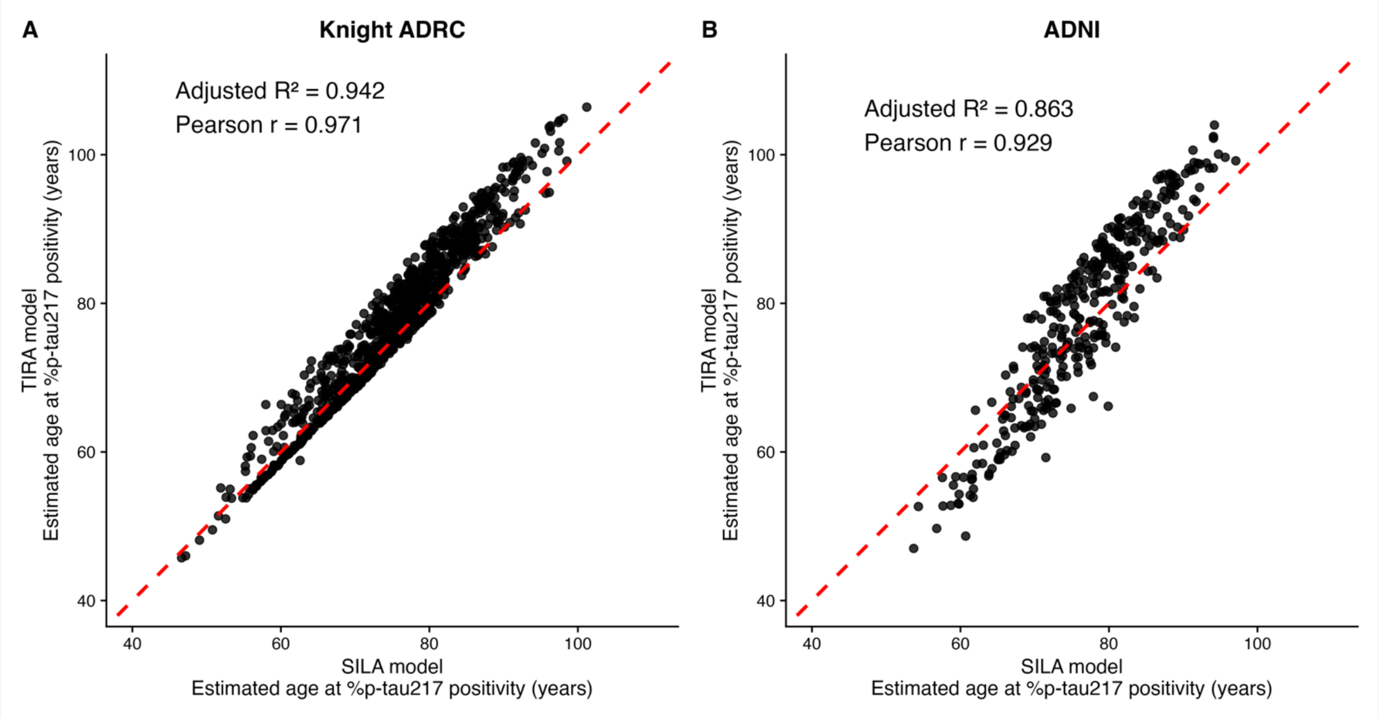

Supplementary Figure 4. Probability of remaining cognitively unimpaired from AD is related to age at plasma %p-tau217 positivity. For individuals who were cognitively unimpaired at baseline in the Knight ADRC (**A, B**) or ADNI (**C, D**) cohorts, Cox models evaluated the probability of remaining cognitively unimpaired from AD as a function of age, stratified by the age at %p-tau217 positivity based on TIRA (**A, C**) or SILA (**B, D**).

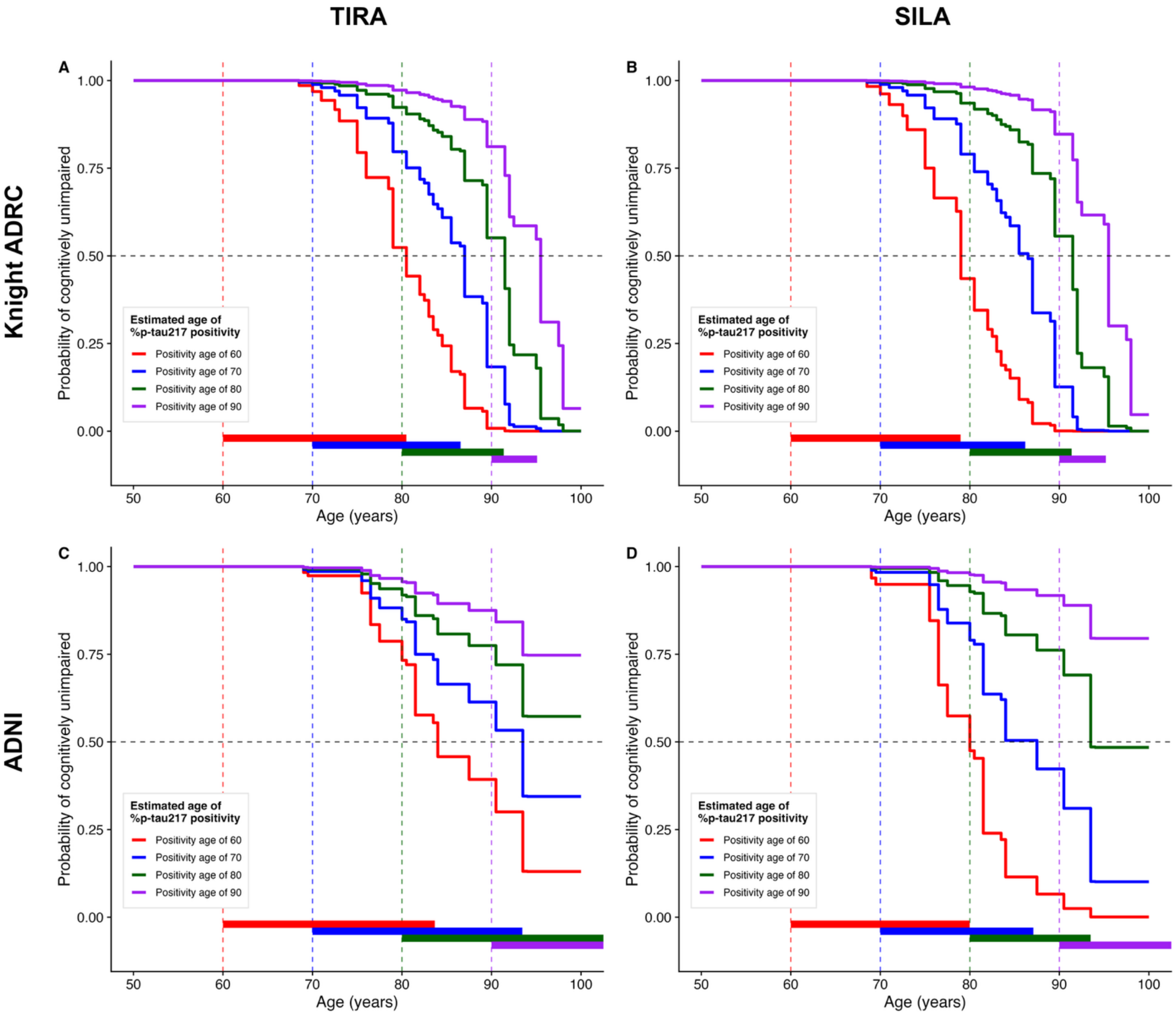

Supplementary Figure 5. Probability of remaining cognitively unimpaired from AD is related to age at plasma %p-tau217 positivity. For all individuals, including those who were cognitively impaired at baseline in the Knight ADRC (**A, B**) or ADNI (**C, D**) cohorts, Cox models evaluated the probability of remaining cognitively unimpaired from AD as a function of age, stratified by the age at %p-tau217 positivity based on TIRA (**A, C**) or SILA (**B, D**).

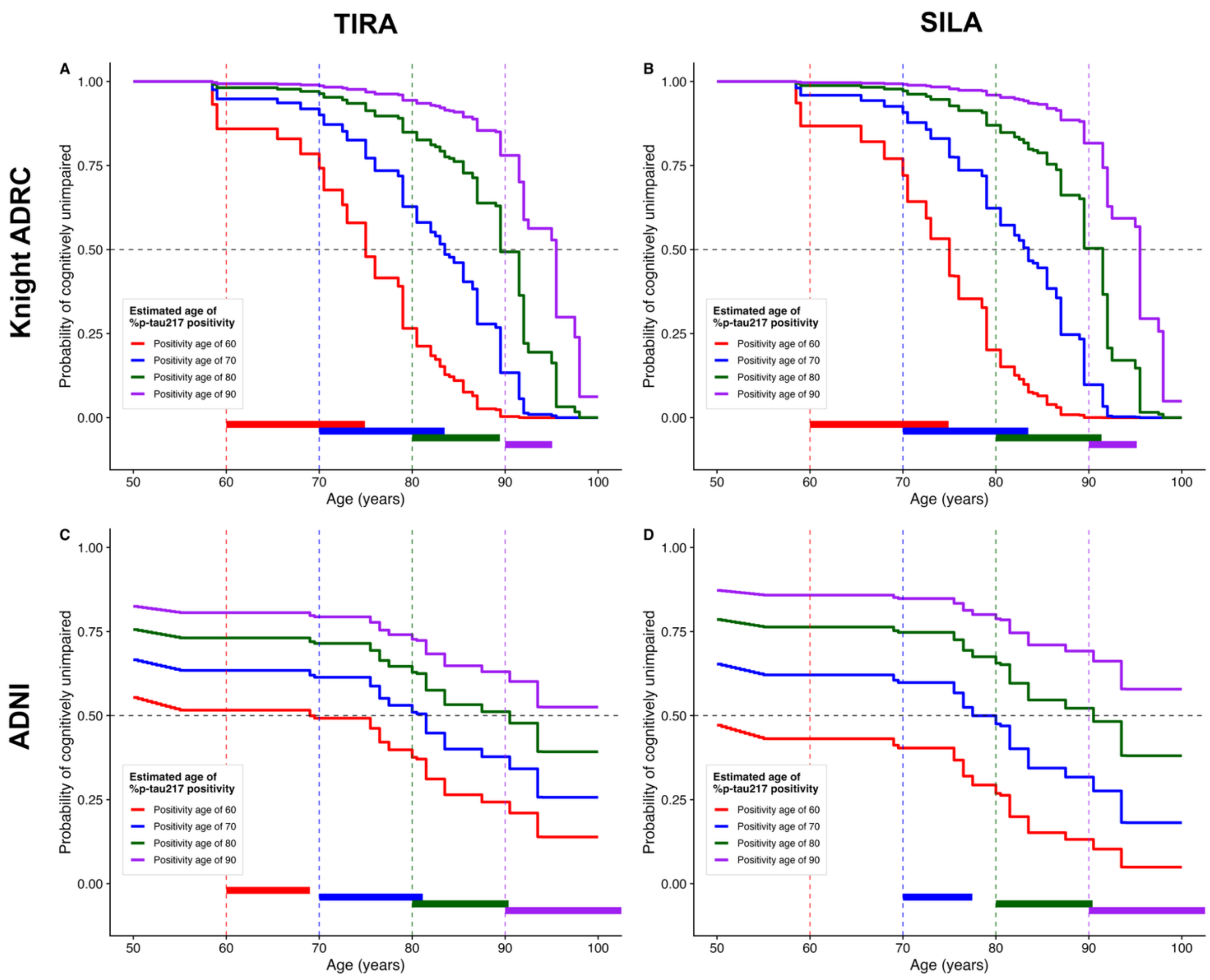

Supplementary Figure 6. Models for time from plasma %p-tau217 positivity to symptom onset based on estimated age at plasma %p-tau217 positivity. Individuals were included who were initially cognitively unimpaired but had a typical AD syndrome at their last assessment and developed symptoms after estimated plasma %p-tau217 positivity. Age at %p-tau217 positivity was estimated using TIRA (**A**) or SILA (**B**) models in the Knight ADRC (green points) or ADNI (black points) cohorts. The time interval was calculated as the difference between age at first diagnosis of cognitive impairment due to AD and the estimated age at %p-tau217 positivity. Linear regression lines with 95% confidence intervals are shown for each dataset, with black lines representing ADNI data and green lines representing Knight ADRC data.

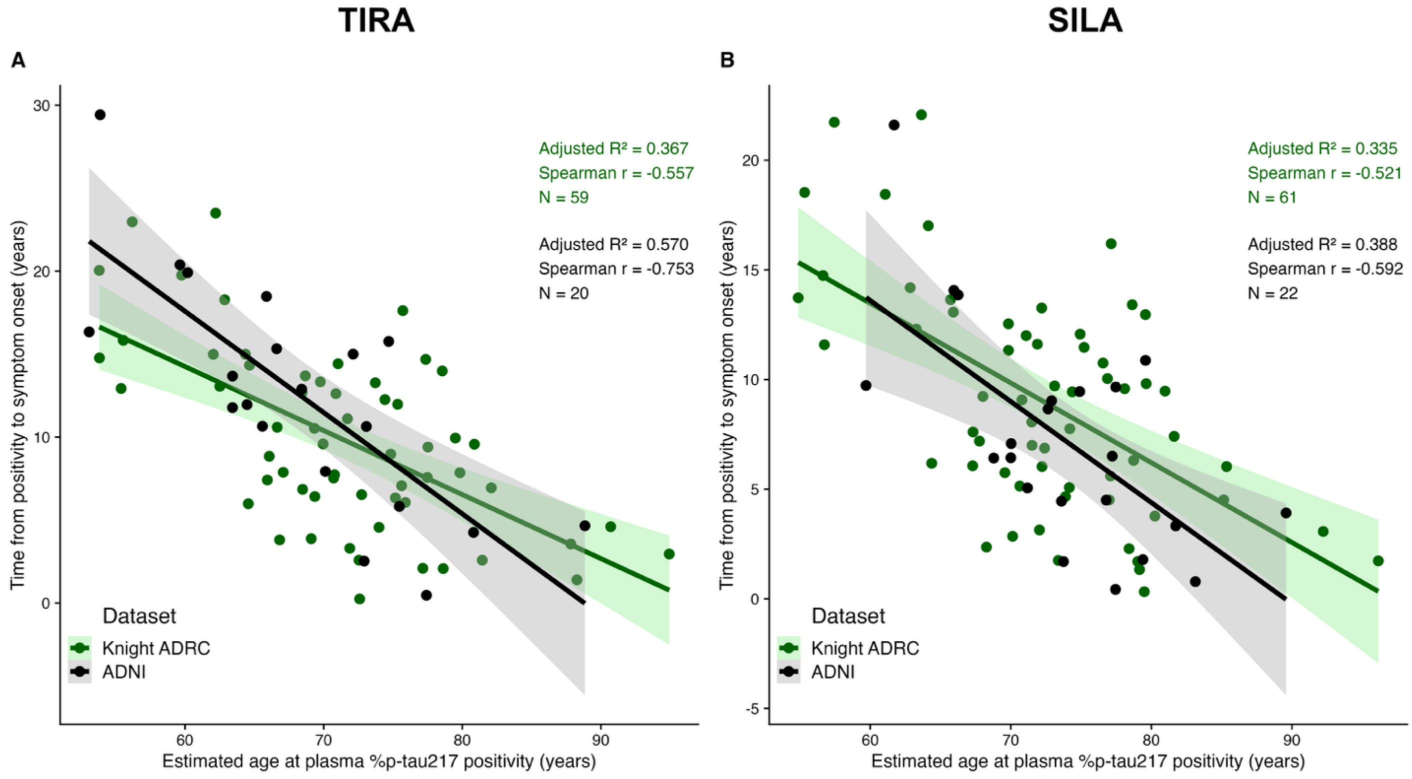

Supplementary Figure 7. Models for age at symptom onset based on estimated age at plasma %p-tau217 positivity. Individuals were included who were initially cognitively unimpaired but had a typical AD syndrome at their last assessment. Individuals who developed symptoms before estimated plasma %p-tau217 positivity, who were excluded in the models described in **Figure 4**, were included in these models. Age at %p-tau217 positivity was estimated using TIRA (**A**) or SILA (**B**) models in the Knight ADRC (green points) or ADNI (black points) cohorts. Linear regression lines with 95% confidence intervals are shown for each dataset, with black lines representing ADNI data and green lines representing Knight ADRC data.

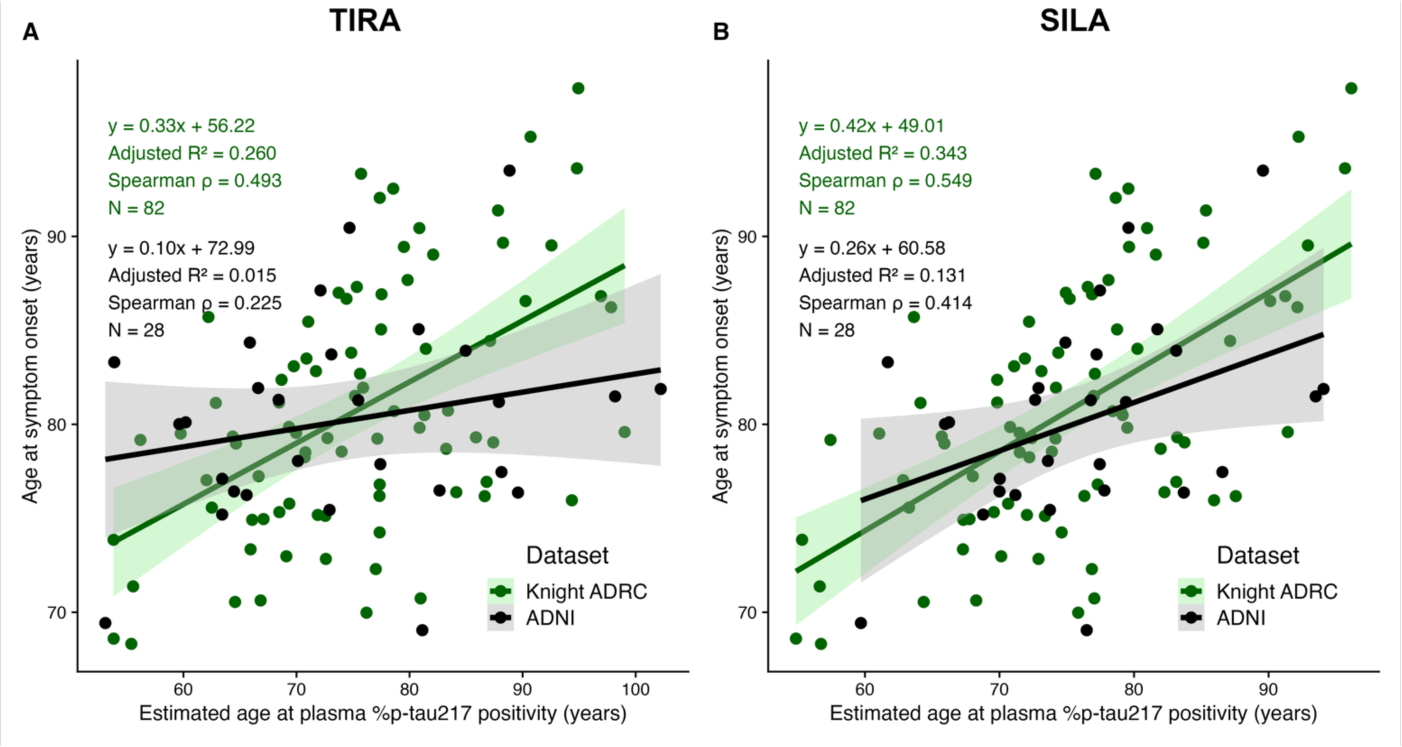

Supplementary Figure 8. Clinical diagnosis as a function of estimated age at plasma %p-tau217 positivity and years from %p-tau217 positivity for initially cognitively unimpaired individuals. In the Knight ADRC (**A, B**) and ADNI (**C, D**) cohorts, age at plasma %p-tau217 positivity was estimated using either the TIRA (**A, C**) or SILA (**B, D**) models. Each row represents the longitudinal clinical diagnoses for one individual by estimated years from %p-tau217 positivity (x-axis). Individuals are sorted vertically by estimated age at %p-tau217 positivity (y-axis). The point color denotes the clinical diagnosis: blue was cognitively unimpaired at the assessment; red (AD syndrome/biomarker positive) was cognitively impaired at the assessment and had a diagnosis of symptomatic AD at their last assessment with symptoms starting after %p-tau217 positivity; purple (AD syndrome/biomarker negative) was cognitively impaired at the assessment and had a diagnosis of symptomatic AD at their last assessment with symptoms starting before %p-tau217 positivity; orange (non-AD syndrome) was cognitively impaired and had a non-AD diagnosis at their last assessment. Vertical dashed lines at 0 represent the estimated time of %p-tau217 positivity. Brown lines indicate the estimated relationship between %p-tau217 positivity age and predicted symptom onset based on the Knight ADRC models in **Figure 4**.

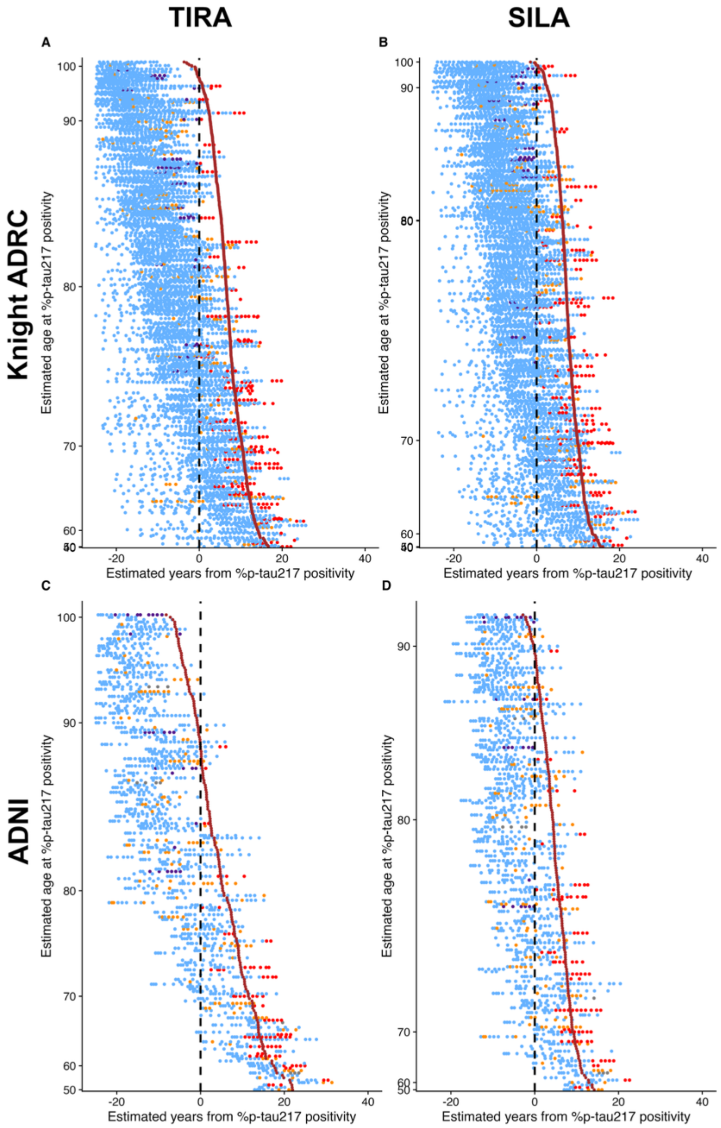

Supplementary Figure 9. Relationships between cognitive impairment and %p-tau217 positivity by SILA using the Knight ADRC dataset. For initially cognitively unimpaired individuals, three groups were examined: red (AD syndrome/biomarker positive) had a diagnosis of symptomatic AD at their last assessment with symptoms starting after %p-tau217 positivity; purple (AD syndrome/biomarker negative) had a diagnosis of symptomatic AD at their last assessment with symptoms starting before %p-tau217 positivity; orange (non-AD syndrome) had a non-AD diagnosis at their last assessment. Kaplan-Meier curves show the probability for each group of remaining cognitively unimpaired as a function of time from first positive %p-tau217 collection (**A, D**), estimated years from %p-tau217 positivity (**B, E**), or estimated years from symptom onset (**C, F**). Density plots and points beneath Kaplan Meier curves (**A-C**) represent the onset of symptoms for individuals in each group. For the red group (AD syndrome/biomarker positive), Kaplan-Meier curves are shown for individuals binned by estimated age of %p-tau217 positivity (blue, <70 years; cyan, 70-80 years; green, ≥ 80 years) (**D-F**).

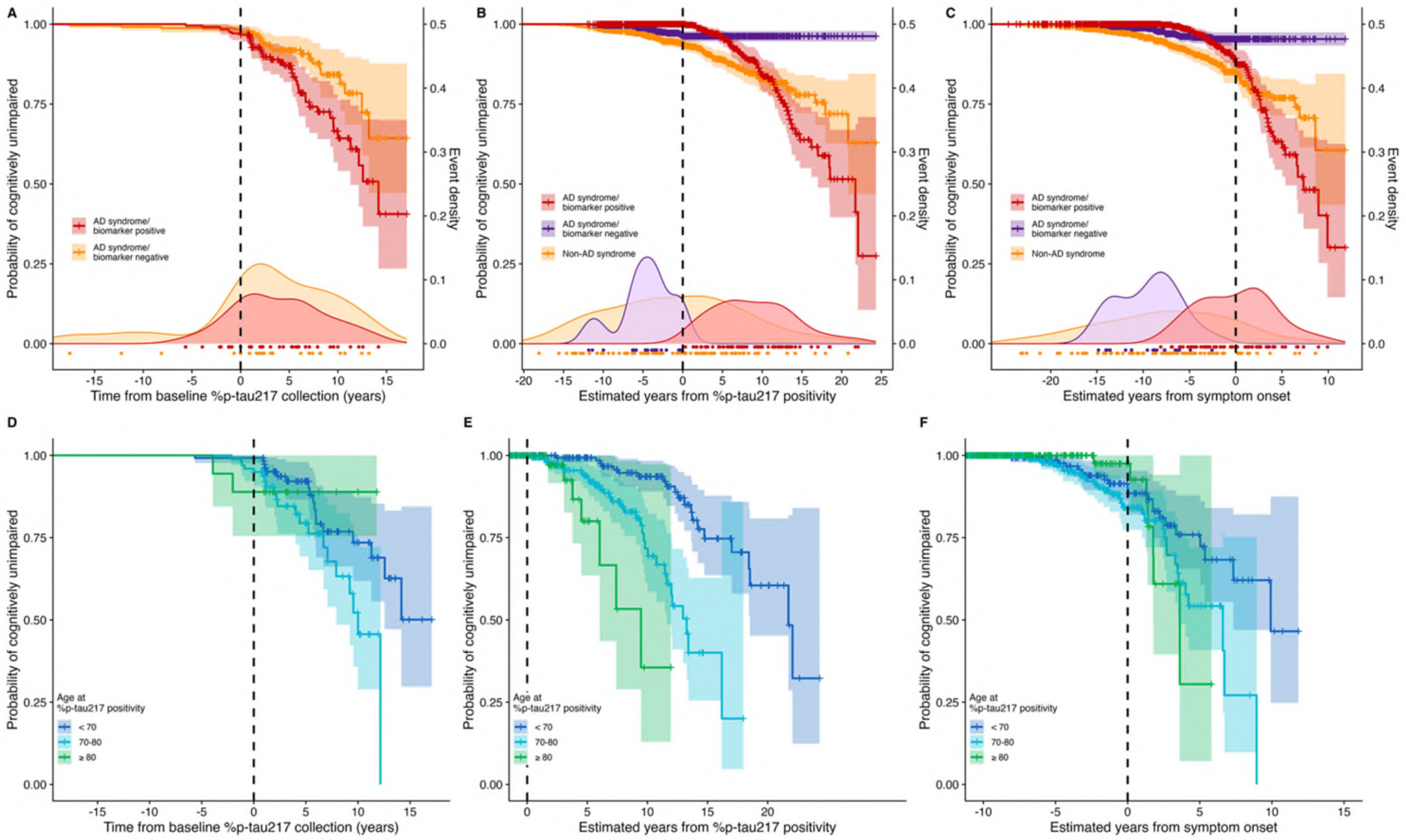

Supplementary Figure 10. Relationships between cognitive impairment and %p-tau217 positivity by TIRA using the ADNI dataset. For initially cognitively unimpaired individuals, three groups were examined: red (AD syndrome/biomarker positive) had a diagnosis of symptomatic AD at their last assessment with symptoms starting after %p-tau217 positivity; purple (AD syndrome/biomarker negative) had a diagnosis of symptomatic AD at their last assessment with symptoms starting before %p-tau217 positivity; orange (non-AD syndrome) had a non-AD diagnosis at their last assessment. Kaplan-Meier curves show the probability for each group of remaining cognitively unimpaired as a function of time from first positive %p-tau217 collection (**A, D**), estimated years from %p-tau217 positivity (**B, E**), or estimated years from symptom onset (**C, F**). Density plots and points beneath Kaplan Meier curves (**A-C**) represent the onset of symptoms for individuals in each group. For the red group (AD syndrome/biomarker positive), Kaplan-Meier curves are shown for individuals binned by estimated age of %p-tau217 positivity (blue, <70 years; cyan, 70-80 years; green, ≥ 80 years) (**D-F**).

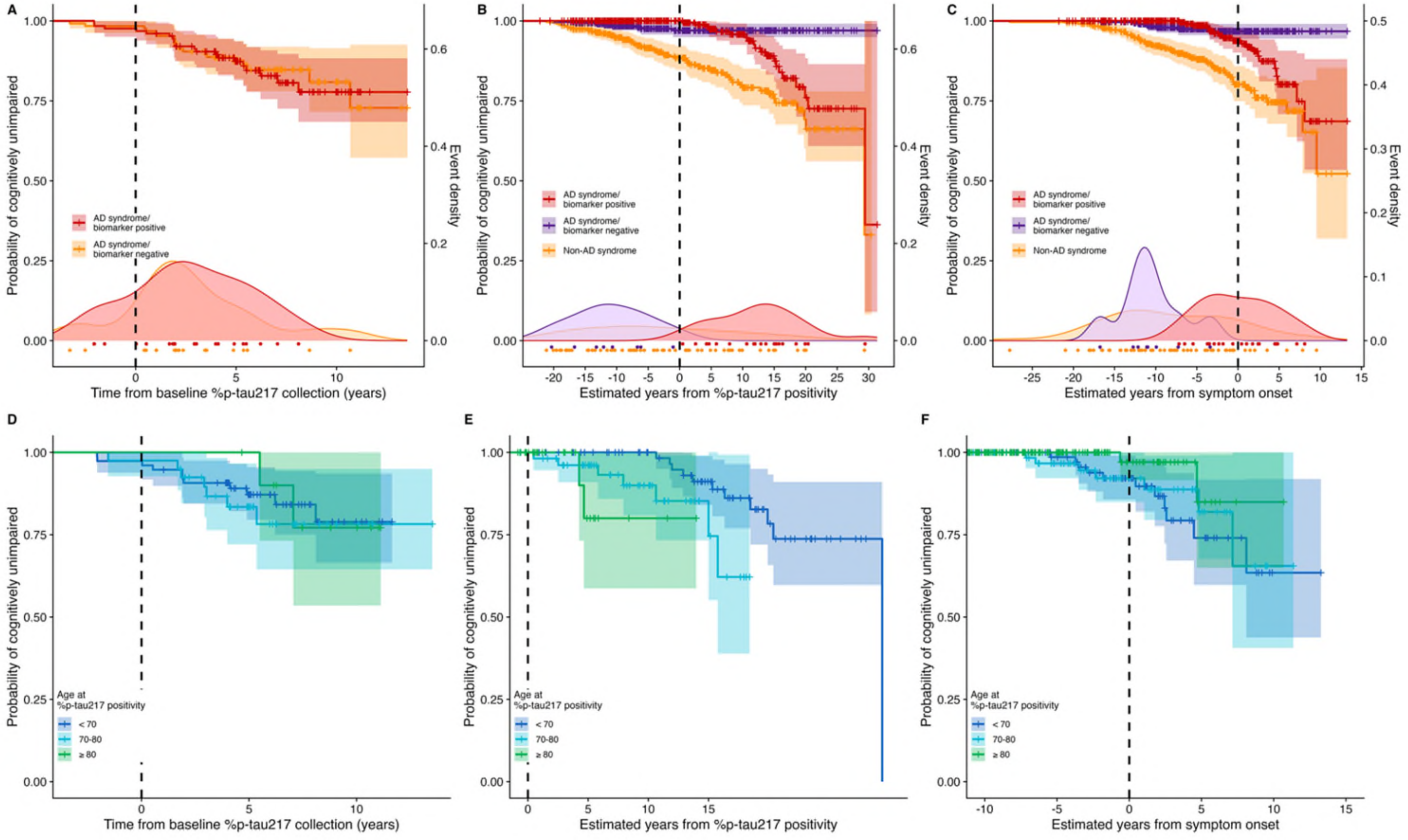

Supplementary Figure 11. Relationships between cognitive impairment and %p-tau217 positivity by SILA using the ADNI dataset. For initially cognitively unimpaired individuals, three groups were examined: red (AD syndrome/biomarker positive) had a diagnosis of symptomatic AD at their last assessment with symptoms starting after %p-tau217 positivity; purple (AD syndrome/biomarker negative) had a diagnosis of symptomatic AD at their last assessment with symptoms starting before %p-tau217 positivity; orange (non-AD syndrome) had a non-AD diagnosis at their last assessment. Kaplan-Meier curves show the probability for each group of remaining cognitively unimpaired as a function of time from first positive %p-tau217 collection (**A, D**), estimated years from %p-tau217 positivity (**B, E**), or estimated years from symptom onset (**C, F**). Density plots and points beneath Kaplan Meier curves (**A-C**) represent the onset of symptoms for individuals in each group. For the red group (AD syndrome/biomarker positive), Kaplan-Meier curves are shown for individuals binned by estimated age of %p-tau217 positivity (blue, <70 years; cyan, 70-80 years; green, ≥ 80 years) (**D-F**).

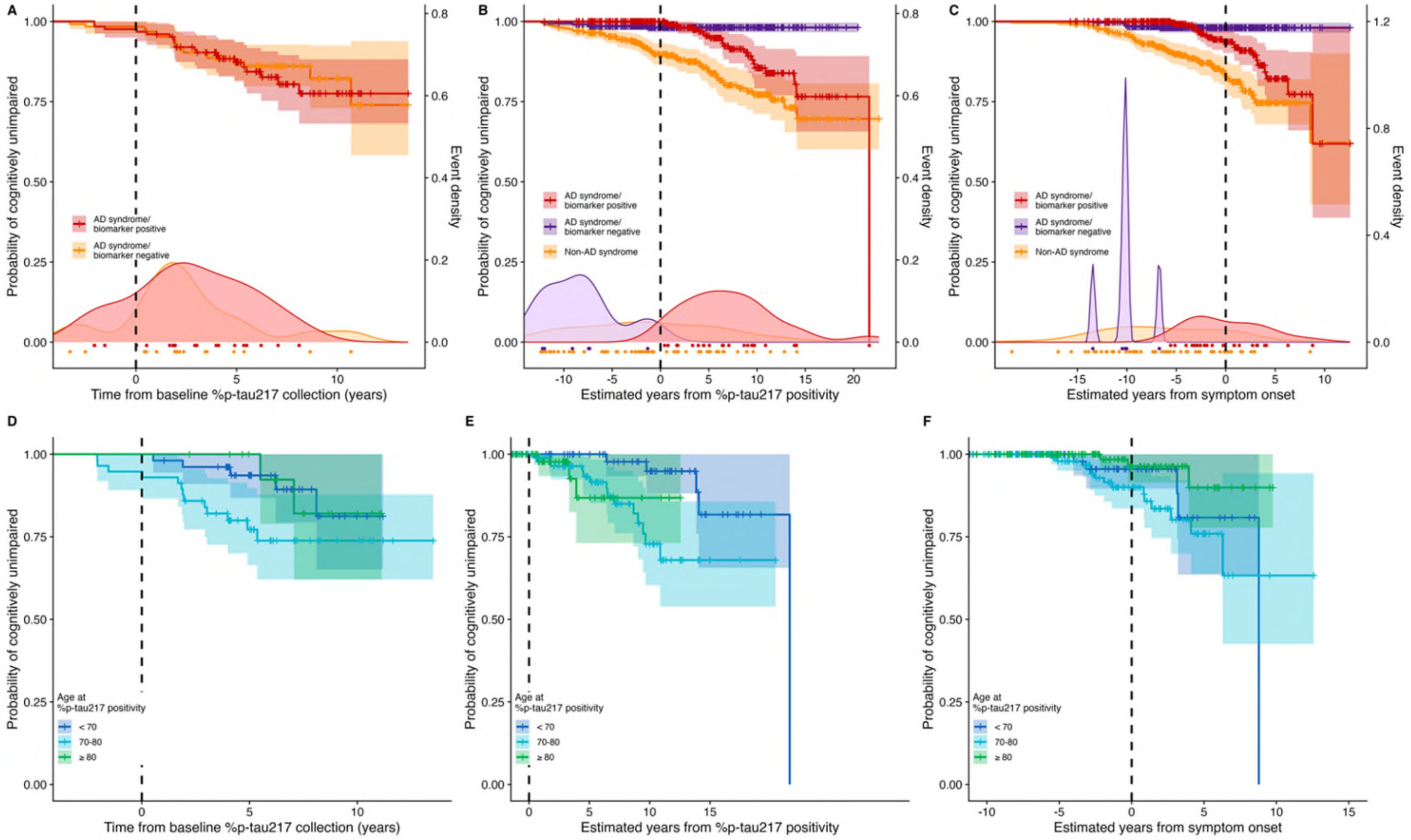

Supplementary Figure 12. C2N Diagnostics' PrecivityAD2 plasma p-tau217 and symptom onset models based on estimated years from p-tau217 positivity. Longitudinal plasma p-tau217 data from ADNI is shown as a function of age (**A**) or estimated years from p-tau217 positivity by TIRA (**B**) or SILA clock models (**C**). Thick black lines represent the clock models; red lines represent individuals with at least one plasma p-tau217>2.34 pg/ml and grey lines represent individuals with no plasma p-tau217>2.34 pg/ml. The horizontal black dashed lines represent the plasma p-tau217 threshold of 2.34 pg/ml[1]. Models for age at symptom onset included individuals who were initially cognitively unimpaired but had a typical AD syndrome at their last assessment and developed symptoms after estimated plasma p-tau217 positivity. Age at p-tau217 positivity was estimated using TIRA (**D**) or SILA (**E**) models. Linear regression lines with 95% confidence intervals are shown.

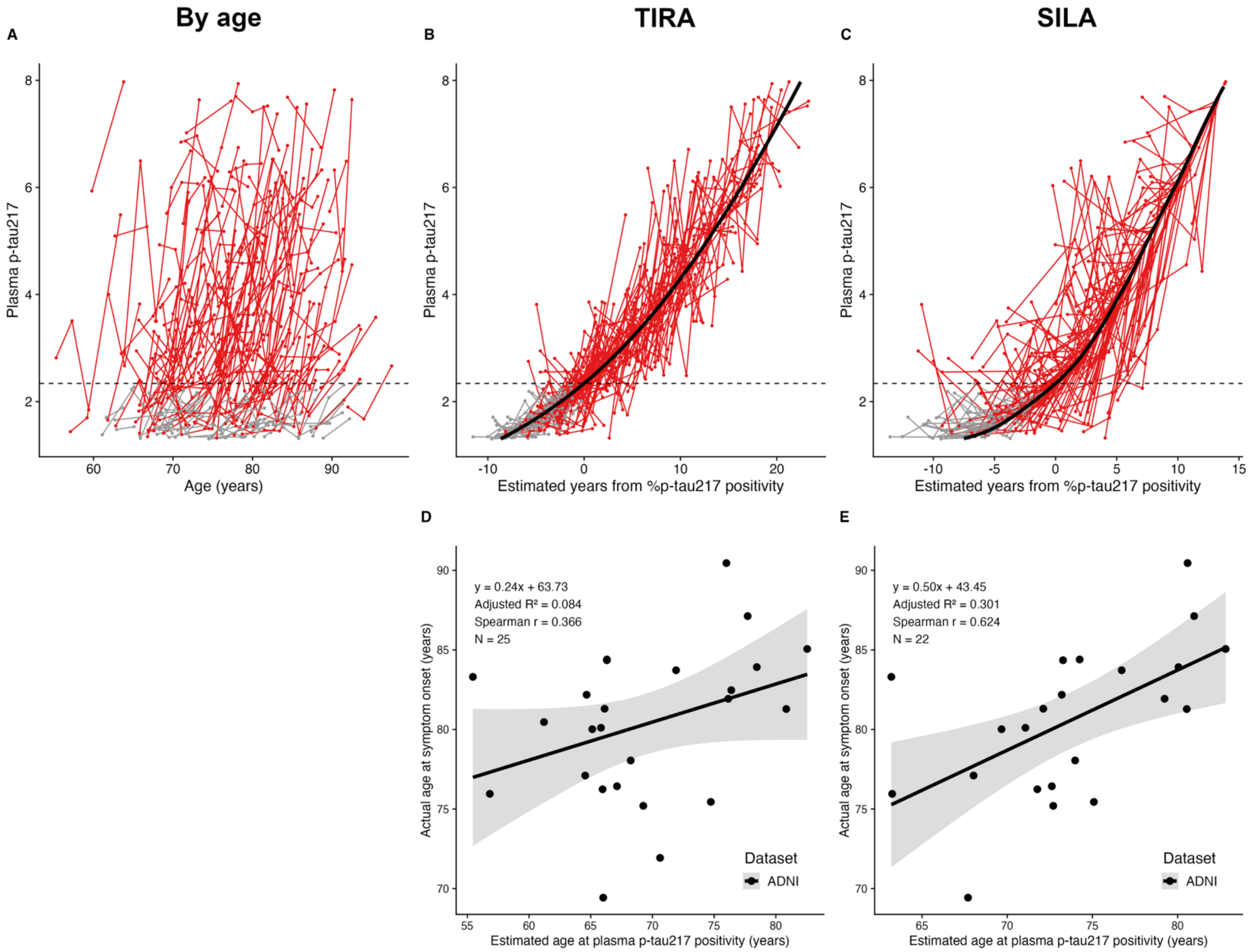

Supplementary Figure 13. Fujirebio Diagnostics' Lumipulse plasma p-tau217 and symptom onset models based on estimated years from p-tau217 positivity. Longitudinal plasma p-tau217 data from ADNI is shown as a function of age (**A**) or estimated years from p-tau217 positivity by TIRA (**B**) or SILA clock models (**C**). Thick black lines represent the clock models; red lines represent individuals with at least one plasma p-tau217>0.158 pg/ml and grey lines represent individuals with no plasma p-tau217>0.158 pg/ml. The horizontal black dashed lines represent the plasma p-tau217 threshold of 0.158 pg/ml[1]. Models for age at symptom onset included individuals who were initially cognitively unimpaired but had a typical AD syndrome at their last assessment and developed symptoms after estimated plasma p-tau217 positivity. Age at p-tau217 positivity was estimated using TIRA (**D**) or SILA (**E**) models. Linear regression lines with 95% confidence intervals are shown.

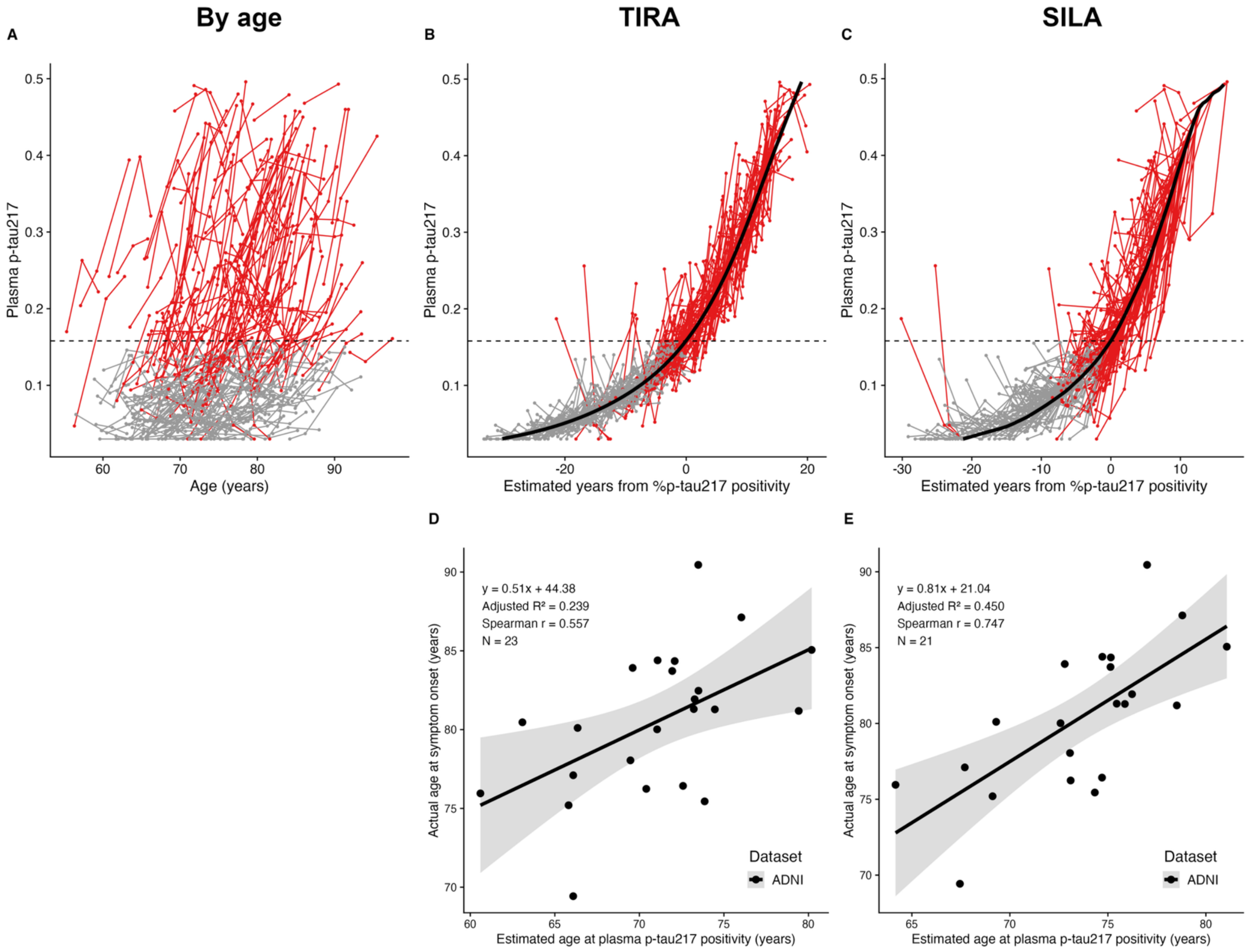

Supplementary Video 1. Animation of C2N Diagnostics' PrecivityAD2 plasma %p-tau217 trajectories transitioning between plots versus age and years since plasma %p-tau217 positivity. The animation shows longitudinal plasma %p-tau217 data plotted as a function of age and years since plasma positivity. Red lines represent individuals with at least one plasma %p-tau217>4.06%, grey lines represent individuals with no plasma %p-tau217>4.06%, and thick black lines represent the clock models.

Supplementary Video 2. Animation of C2N Diagnostics' PrecivityAD2 plasma p-tau217 trajectories transitioning between plots versus age and years since plasma p-tau217 positivity. The animation shows longitudinal plasma p-tau217 data plotted as a function of age and years since plasma positivity. Red lines represent individuals with at least one plasma p-tau217>2.34 pg/ml, grey lines represent individuals with no plasma p-tau217>2.34 pg/ml, and thick black lines represent the clock models.

Supplementary Video 3. Animation of Fujirebio Diagnostics' Lumipulse plasma p-tau217/Aβ42 trajectories transitioning between plots versus age and years since plasma p-tau217/Aβ42 positivity. The animation shows longitudinal plasma p-tau217/Aβ42 data plotted as a function of age and years since plasma positivity. Red lines represent individuals with at least one plasma p-tau217/Aβ42>0.006312, grey lines represent individuals with no plasma p-tau217/Aβ42>0.006312, and thick black lines represent the clock models.

Supplementary Video 4. Animation of Fujirebio Diagnostics' Lumipulse plasma p-tau217 trajectories transitioning between plots versus age and years since plasma p-tau217 positivity. The animation shows longitudinal plasma p-tau217 data plotted as a function of age and years since plasma positivity. Red lines represent individuals with at least one plasma p-tau217>0.158 pg/ml, grey lines represent individuals with no plasma p-tau217>0.158 pg/ml, and thick black lines represent the clock models.

Supplementary Video 5. Animation of Janssen's LucentAD Quanterix plasma p-tau217 trajectories transitioning between plots versus age and years since plasma positivity. The animation shows longitudinal plasma p-tau217 data plotted as a function of age and years since plasma positivity. Red lines represent individuals with at least one plasma p-tau217>0.0615 pg/ml, grey lines represent individuals with no plasma p-tau217>0.0615 pg/ml, and thick black lines represent the clock models.

Supplementary Video 6. Animation of ALZpath's Quanterix plasma p-tau217 trajectories transitioning between plots versus age and years since plasma p-tau217 positivity. The animation shows longitudinal plasma p-tau217 data plotted as a function of age and years since plasma positivity. Red lines represent individuals with at least one plasma p-tau217>0.444 pg/ml, grey lines represent individuals with no plasma p-tau217>0.444 pg/ml, and thick black lines represent the clock models.
