# Supplementary material for: Predicting onset of symptomatic Alzheimerʼs disease with plasma p-tau217 clocks
Source: Nat Med. 2026 Feb 19;32(3):1085–94. doi: 10.1038/s41591-026-04206-y (PMC13004683; doi:10.1038/s41591-026-04206-y)
Supplement: Supplementary file 2 — Reporting Summary [file 41591_2026_4206_MOESM2_ESM.pdf]

nature portfolio

Corresponding author(s): Suzanne E. Schindler

Last updated by author(s): Dec 13, 2025

Reporting Summary

Nature Portfolio wishes to improve the reproducibility of the work that we publish. This form provides structure for consistency and transparency in reporting. For further information on Nature Portfolio policies, see our Editorial Policies and the Editorial Policy Checklist.

Statistics

For all statistical analyses, confirm that the following items are present in the figure legend, table legend, main text, or Methods section.

n/a	Confirmed	
☐	☒	The exact sample size (*n*) for each experimental group/condition, given as a discrete number and unit of measurement
☐	☒	A statement on whether measurements were taken from distinct samples or whether the same sample was measured repeatedly
☐	☒	The statistical test(s) used AND whether they are one- or two-sided<br>*Only common tests should be described solely by name; describe more complex techniques in the Methods section.*
☐	☒	A description of all covariates tested
☐	☒	A description of any assumptions or corrections, such as tests of normality and adjustment for multiple comparisons
☐	☒	A full description of the statistical parameters including central tendency (e.g. means) or other basic estimates (e.g. regression coefficient) AND variation (e.g. standard deviation) or associated estimates of uncertainty (e.g. confidence intervals)
☐	☒	For null hypothesis testing, the test statistic (e.g. *F*, *t*, *r*) with confidence intervals, effect sizes, degrees of freedom and *P* value noted<br>*Give P values as exact values whenever suitable.*
☒	☐	For Bayesian analysis, information on the choice of priors and Markov chain Monte Carlo settings
☐	☒	For hierarchical and complex designs, identification of the appropriate level for tests and full reporting of outcomes
☐	☒	Estimates of effect sizes (e.g. Cohen's *d*, Pearson's *r*), indicating how they were calculated

Our web collection on statistics for biologists contains articles on many of the points above.

Software and code

Policy information about availability of computer code

Data collection	Plasma %p-tau217 was measured by C2N Diagnostics using liquid chromatography mass spectrometry (LC-MS) based assay. Additional assays used Fujirebio Lumipulse G1200 analyzer and Quanterix Simoa-HD-X analyzer. Clinical assessments used standardized protocols from Knight ADRC and ADNI.
Data analysis	R version 4.4.1 was used for all analyses except SILA models, which used Matlab 2024b. Key R packages included: tidyverse (data manipulation, version 2.0.0), mgcv (GAMs, version 1.9.3), icenReg (interval-censored regression, version 2.0.16), nlme (mixed-effects modeling, version 3.1.168), survival (survival analysis, version 3.8.3), DescTools (concordance correlation, version 0.99.60), doParallel (parallel computing, version 1.0.17). Code available at: https://github.com/WashU-FluidBiomarkers/plasma-ptau217-time

For manuscripts utilizing custom algorithms or software that are central to the research but not yet described in published literature, software must be made available to editors and reviewers. We strongly encourage code deposition in a community repository (e.g. GitHub). See the Nature Portfolio guidelines for submitting code & software for further information.

Data

Policy information about availability of data

All manuscripts must include a data availability statement. This statement should provide the following information, where applicable:

- Accession codes, unique identifiers, or web links for publicly available datasets
- A description of any restrictions on data availability
- For clinical datasets or third party data, please ensure that the statement adheres to our policy

Data from the Knight ADRC can be requested by qualified investigators (knightadrc.wustl.edu/Research/ResourceRequest.htm). Data from ADNI can be requested via the LONI website (adni.loni.usc.edu). Code developed by the authors is available at https://github.com/WashU-FluidBiomarkers/plasma-ptau217-time

Research involving human participants, their data, or biological material

Policy information about studies with human participants or human data. See also policy information about sex, gender (identity/presentation), and sexual orientation and race, ethnicity and racism.

Reporting on sex and gender	Sex was reported as a biological attribute in both cohorts. In the full longitudinal cohort (n=912), 52% were female. Knight ADRC had 54.2% female participants (274/506) and ADNI had 49.3% female participants (200/406). Sex was considered as a covariate in symptom onset models but was not a significant predictor and thus not included in final models. Complete sex distributions are provided in Supplementary Tables 1, 2, 6, and 7 for all analysis cohorts.
Reporting on race, ethnicity, or other socially relevant groupings	Participants largely identified as non-Hispanic White, which may limit the generalizability of these models to other groups, especially groups with different rates of non-AD co-pathologies. This limitation in demographic diversity is acknowledged in the manuscript's discussion section.
Population characteristics	Detailed demographic characteristics are provided in Supplementary Tables 1, 2, 6, and 7. Key baseline characteristics include: median age 69.8 years (Knight ADRC: 67.7 years, ADNI: 72.7 years), 35.1% APOE ε4 carriers, median education 16 years. Cognitive status varied by cohort: Knight ADRC had 8.5% cognitively impaired at baseline vs 48.3% in ADNI. Follow-up time ranged from 4-14 years with multiple longitudinal assessments.
Recruitment	Participants were community-dwelling older adults enrolled in established longitudinal aging studies. Knight ADRC participants were recruited through the Knight Alzheimer Disease Research Center at Washington University, focused on characterizing preclinical AD transitions. ADNI participants were recruited through the multi-center Alzheimer's Disease Neuroimaging Initiative, representing a collaborative public-private partnership. Both cohorts used standardized protocols for clinical and biomarker assessments. No specific selection biases are reported, though the predominantly non-Hispanic White demographic may limit generalizability.
Ethics oversight	All participants provided written informed consent. The study followed STROBE requirements for observational studies. Research was conducted through established IRB-approved protocols at the Knight Alzheimer Disease Research Center (Washington University) and the Alzheimer's Disease Neuroimaging Initiative consortium.

Note that full information on the approval of the study protocol must also be provided in the manuscript.

Field-specific reporting

Please select the one below that is the best fit for your research. If you are not sure, read the appropriate sections before making your selection.

☒ Life sciences ☐ Behavioural & social sciences ☐ Ecological, evolutionary & environmental sciences

For a reference copy of the document with all sections, see nature.com/documents/nr-reporting-summary-flat.pdf

Life sciences study design

All studies must disclose on these points even when the disclosure is negative.

Sample size	Sample sizes were determined by the availability of participants with longitudinal plasma %p-tau217 measurements in two established cohorts. The Knight ADRC clock cohort included 258 individuals and the ADNI clock cohort included 345 individuals. For symptom onset models, sample sizes were: Knight ADRC (59-61 individuals) and ADNI (20-22 individuals). No formal power calculations were performed as this was an exploratory analysis using available longitudinal data from established aging cohorts. Sample sizes are reported for each analysis in the main text and Supplementary Tables 1 and 2.
Data exclusions	Participants were excluded if they had fewer than two plasma %p-tau217 measurements at least one year apart. For clock model development, analysis was restricted to individuals with plasma %p-tau217 values between 1.06-10.45, representing the range with consistent rates of change identified through variance analysis. Values outside this range showed high variance or sparse data that would make time estimates unstable. For symptom onset models, individuals were excluded if they were cognitively impaired at baseline, had transient cognitive impairment that resolved, or had non-AD diagnoses at final assessment.
Replication	The study employed multiple forms of replication: (1) Cross-cohort validation using two independent datasets (Knight ADRC and ADNI), (2) Cross-validation where models trained in one cohort were tested in the other, achieving moderate associations (adjusted R^2 0.463-0.577), (3)

	Two different mathematical approaches (TIRA and SILA) that yielded similar results, providing methodological replication, and (4) Validation across multiple plasma p-tau217 assays (5 different commercial assays tested).
Randomization	Not applicable. This was an observational longitudinal study of aging cohorts. Participants were not randomized to groups. Clinical assessments and plasma collections followed standardized protocols in both cohorts at predetermined intervals.
Blinding	Clinicians performing clinical assessments and determining cognitive diagnoses were blinded to biomarker results. Clinical syndrome determinations (AD vs non-AD) were made based solely on clinical presentation and established diagnostic criteria without knowledge of plasma %p-tau217 values or other biomarker results.

Reporting for specific materials, systems and methods

We require information from authors about some types of materials, experimental systems and methods used in many studies. Here, indicate whether each material, system or method listed is relevant to your study. If you are not sure if a list item applies to your research, read the appropriate section before selecting a response.

Materials & experimental systems

n/a	Involved in the study
☒	☐ Antibodies
☒	☐ Eukaryotic cell lines
☒	☐ Palaeontology and archaeology
☒	☐ Animals and other organisms
☐	☒ Clinical data
☒	☐ Dual use research of concern
☒	☐ Plants

Methods

n/a	Involved in the study
☒	☐ ChIP-seq
☒	☐ Flow cytometry
☒	☐ MRI-based neuroimaging

Clinical data

Policy information about clinical studies

All manuscripts should comply with the ICMJE guidelines for publication of clinical research and a completed CONSORT checklist must be included with all submissions.

Clinical trial registration	Not applicable. This was an observational longitudinal study using existing cohorts (Knight ADRC and ADNI), not a clinical trial requiring registration. Both studies follow established longitudinal protocols approved by their respective institutional review boards.
Study protocol	Full study protocols are available through the respective cohort websites. Knight ADRC protocols can be accessed through https://knightadrc.wustl.edu/Research/ResourceRequest.htm. ADNI protocols are available at https://adni.loni.usc.edu. Both cohorts use standardized protocols for clinical assessments, biomarker collection, and longitudinal follow-up that have been previously published and are publicly documented.
Data collection	Data were collected at two sites: Knight Alzheimer Disease Research Center at Washington University in St. Louis (single-site cohort) and multiple centers participating in the Alzheimer's Disease Neuroimaging Initiative (ADNI, multi-center consortium). Plasma samples were collected during routine study visits following standardized protocols. Knight ADRC participants had plasma collected over a median of 7.1 years (2012-2024 timeframe), while ADNI participants had plasma collected over a median of 5.0 years (2005-2021 timeframe). Clinical assessments were conducted at regular intervals using standardized protocols including Clinical Dementia Rating (CDR) evaluations and neurological examinations.
Outcomes	Primary outcome was age at onset of symptomatic Alzheimer disease, defined as the first clinical assessment when initially cognitively unimpaired (CDR=0) individuals with positive AD biomarkers developed cognitive impairment (CDR>0) with an AD syndrome. Secondary outcomes included probability of developing symptomatic AD over time and cognitive impairment as measured by the Preclinical Alzheimer Cognitive Composite (PACC). Clinical syndrome determinations were made by clinicians blinded to biomarker results using established diagnostic criteria. All assessments followed standardized protocols with interval-censored timing to account for variable assessment intervals.

Plants

Seed stocks	n/a
Novel plant genotypes	n/a
Authentication	n/a
