# Supplementary figures and images for: Predicting onset of symptomatic Alzheimerʼs disease with plasma p-tau217 clocks
Source: Nat Med. 2026 Feb 19;32(3):1085–94. doi: 10.1038/s41591-026-04206-y (PMC13004683; doi:10.1038/s41591-026-04206-y)

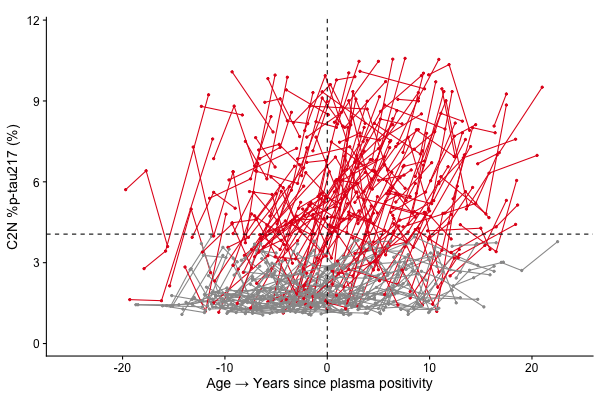

Supplement: Supplementary file 4 — Animation of C2N Diagnostics’ PrecivityAD2 plasma %p-tau217 trajectories transitioning between plots versus age and years since plasma %p-tau217 positivity. The animation shows longitudinal plasma %p-tau217 data plotted as a function of age and years since plasma positivity. Red lines represent individuals with at least one plasma %p-tau217 > 4.06%; gray lines represent individuals with no plasma %p-tau217 > 4.06%; and thick black lines represent the clock models. [file 41591_2026_4206_MOESM4_ESM.gif]

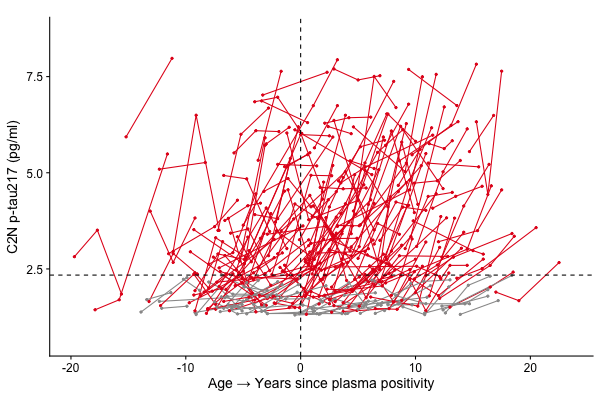

Supplement: Supplementary file 5 — Animation of C2N Diagnostics’ PrecivityAD2 plasma p-tau217 trajectories transitioning between plots versus age and years since plasma p-tau217 positivity. The animation shows longitudinal plasma p-tau217 data plotted as a function of age and years since plasma positivity. Red lines represent individuals with at least one plasma p-tau217 > 2.34 pg ml−1; gray lines represent individuals with no plasma p-tau217 > 2.34 pg ml−1; and thick black lines represent the clock models. [file 41591_2026_4206_MOESM5_ESM.gif]

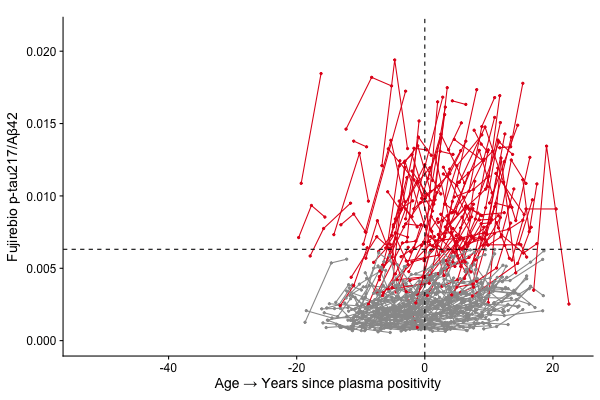

Supplement: Supplementary file 6 — Animation of Fujirebio Diagnostics’ Lumipulse plasma p-tau217/Aβ42 trajectories transitioning between plots versus age and years since plasma p-tau217/Aβ42 positivity. The animation shows longitudinal plasma p-tau217/Aβ42 data plotted as a function of age and years since plasma positivity. Red lines represent individuals with at least one plasma p-tau217/Aβ42 > 0.006312; gray lines represent individuals with no plasma p-tau217/Aβ42 > 0.006312; and thick black lines represent the clock models. [file 41591_2026_4206_MOESM6_ESM.gif]

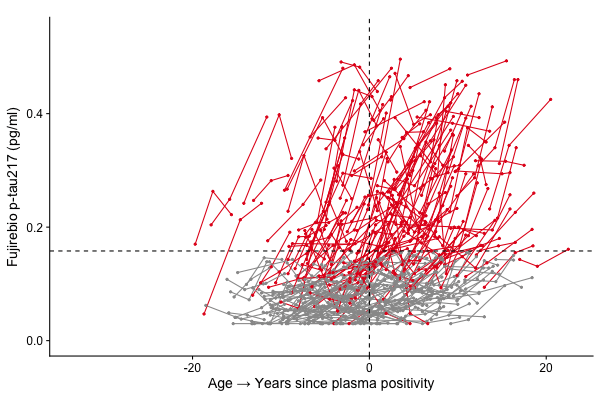

Supplement: Supplementary file 7 — Animation of Fujirebio Diagnostics’ Lumipulse plasma p-tau217 trajectories transitioning between plots versus age and years since plasma p-tau217 positivity. The animation shows longitudinal plasma p-tau217 data plotted as a function of age and years since plasma positivity. Red lines represent individuals with at least one plasma p-tau217 > 0.158 pg ml−1; gray lines represent individuals with no plasma p-tau217 > 0.158 pg ml−1; and thick black lines represent the clock models. [file 41591_2026_4206_MOESM7_ESM.gif]

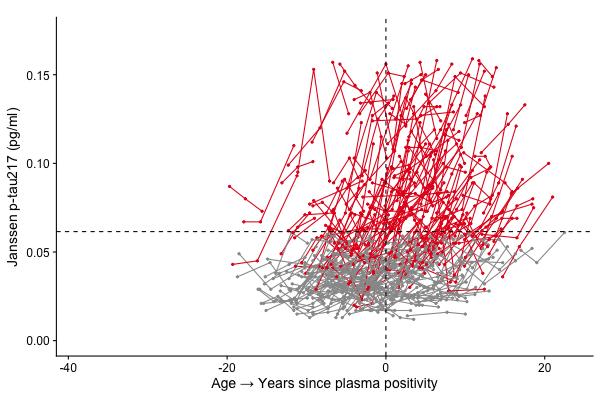

Supplement: Supplementary file 8 — Animation of Janssen’s LucentAD Quanterix plasma p-tau217 trajectories transitioning between plots versus age and years since plasma positivity. The animation shows longitudinal plasma p-tau217 data plotted as a function of age and years since plasma positivity. Red lines represent individuals with at least one plasma p-tau217 > 0.0615 pg ml−1; gray lines represent individuals with no plasma p-tau217 > 0.0615 pg ml−1; and thick black lines represent the clock models. [file 41591_2026_4206_MOESM8_ESM.gif]

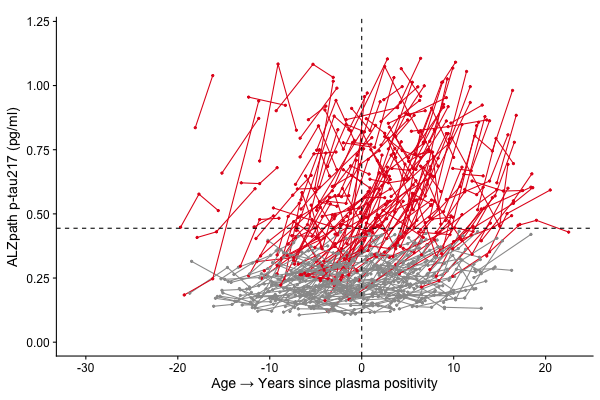

Supplement: Supplementary file 9 — Animation of ALZpath’s Quanterix plasma p-tau217 trajectories transitioning between plots versus age and years since plasma p-tau217 positivity. The animation shows longitudinal plasma p-tau217 data plotted as a function of age and years since plasma positivity. Red lines represent individuals with at least one plasma p-tau217 > 0.444 pg ml−1; gray lines represent individuals with no plasma p-tau217 > 0.444 pg ml−1; and thick black lines represent the clock models. [file 41591_2026_4206_MOESM9_ESM.gif]
